# Cathepsin B Is Not an Intrinsic Factor Related to Asparaginase Resistance of the Acute Lymphoblastic Leukemia REH Cell Line

**DOI:** 10.3390/ijms241311215

**Published:** 2023-07-07

**Authors:** Iris Munhoz Costa, Brian Effer, Tales Alexandre Costa-Silva, Chen Chen, Michael F. Ciccone, Adalberto Pessoa, Camila O. dos Santos, Gisele Monteiro

**Affiliations:** 1 Departamento de Tecnologia Bioquímico-Farmacêutica, Faculdade de Ciências Farmacêuticas, Universidade de São Paulo, São Paulo 05508-000, SP, Brazil; irismunhozcosta@gmail.com (I.M.C.); breeiky@hotmail.com (B.E.); tales.costa@ufabc.edu.br (T.A.C.-S.); pessoajr@usp.br (A.P.); 2 Cold Spring Harbor Laboratory, Cold Spring Harbor, New York, NY 11724, USA; chen@cshl.edu (C.C.); ciccone@cshl.edu (M.F.C.); dossanto@cshl.edu (C.O.d.S.); 3 Center of Excellence in Translational Medicine (CEMT) and Scientific and Technological Bioresource Nucleus (BIOREN), Universidad de La Frontera, Temuco 4780000, Chile; 4 Center for Natural and Human Sciences, Federal University of ABC, Santo André 14040-903, SP, Brazil

**Keywords:** asparaginase, cathepsin B, acute lymphoblastic leukemia, treatment resistance, genetic editing

## Abstract

L-Asparaginase (ASNase) is a biopharmaceutical used as an essential drug in the treatment of acute lymphoblastic leukemia (ALL). Yet, some cases of ALL are naturally resistant to ASNase treatment, which results in poor prognosis. The REH ALL cell line, used as a model for studying the most common subtype of ALL, is considered resistant to treatment with ASNase. Cathepsin B (CTSB) is one of the proteases involved in the regulation of in vivo ASNase serum half-life and it has also been associated with the progression and resistance to treatment of several solid tumors. Previous works have shown that, in vitro, ASNase is degraded when incubated with REH cell lysate, which is prevented by a specific CTSB inhibitor, suggesting a function of this protease in the ASNase resistance of REH cells. In this work, we utilized a combination of CRISPR/Cas9 gene targeting and enzymatic measurements to investigate the relevance of CTSB on ASNase treatment resistance in the ALL model cell line. We found that deletion of CTSB in REH ALL cells did not confer ASNase treatment sensitivity, thus suggesting that intrinsic expression of *CTSB* is not a mechanism that drives the resistant nature of these ALL cells to enzymes used as the first-line treatment against leukemia.

## 1. Introduction

L-Asparaginase (ASNase) has been applied as a first-line treatment for acute lymphoblastic leukemia (ALL) since the 1970s until today, as its antitumor activity increases the5 year disease-free survival rate to 90%. Despite the favorable prognosis, ASNase resistance, either intrinsic or acquired, can ultimately lead to discontinuation of its use and, thus, to high morbidity and mortality [1]. Several mechanisms have been described as the basis for ASNase resistance in ALL patients, including the inactivation of enzyme, which may be due to the formation of antibodies, protease-induced degradation, or, most likely, both [2]. In fact, two cysteine proteases have been linked to ASNase degradation: cathepsin B (CTSB) and asparagine endopeptidase (AEP) [3,4,5,6]. It was demonstrated, in vitro, that *Escherichia coli* ASNase is degraded in the presence of both proteases, while *Erwinia chrysanthemi* ASNase is sensitive only to CTSB [4]. Subsequently, an abnormal increase in the half-life of the *E. chrysanthemi* ASNase was observed in a pediatric ALL patient, who had a germline mutation in the coding gene for *CTSB*, producing reduced protease activity [6]. This observation was also confirmed by *CTSB* gene knockout in mice that showed a longer serum ASNase half-life compared to wildtype mice [5].

Changes in the level of CTSB expression have been extensively studied and associated with the processes of proliferation, invasion, and metastasis of cancer cells. The increased expression in solid tumors (e.g., breast, lung, colon, ovary, bladder, pancreas, thyroid, and prostate) has made this protease a prognostic marker for some types of cancer [7,8,9]. For example, it was demonstrated that the chemotherapeutic response in breast cancer using paclitaxel (Taxol^®^) was impaired by cathepsin-expressing macrophages, and the use of cathepsin inhibitor reverted the effect [10]. Additionally, it has been shown that the role of CTSB in oncogenesis can be completely different depending on the tumor type. Animal models with *CTSB* knockout mammary carcinoma showed a reduction in tumor volume and proliferation, while, in animals with squamous cell carcinoma, there was no change in cell proliferation [8].

In hematological tumors, the relationship between CTSB and disease prognosis has already been reported for some leukemia cell lines. Studies have shown that overexpression of *CTSB* in acute myeloid leukemia (AML) cells is related to a poor prognosis, and inhibition of CTSB in these cells contributes to the mechanism of apoptosis [11]. Furthermore, knockout of *CTSB* in AML cells contributed to the inhibition of proliferation and tumorigenesis [12]. In chronic lymphocytic leukemia (CLL) cells, CTSB was observed as a mediator of the apoptotic response during treatment with valproic acid and fludarabine [13]. In the case of ALL, our group showed that ASNases possessing different sensitivity to the action of lysosomal proteases CTSB and AEP result in altered pharmacokinetic and immunological profiles in vivo (health mice model), but equal cytotoxicity against several ALL cell lines in vitro [2]. Therefore, little is known about the relationship between CTSB protease expressed by tumor cells in the treatment of ALL and ASNase sensitivity.

Here, we demonstrated that CTSB endogenous activity of ALL tumor cells is dispensable for ASNase treatment resistance. We utilized the REH cell line, which possesses a phenotype of the most common subtype of ALL [14], expresses CTSB, and is considered resistant to ASNase treatment [4,15]. Using this cell line as model, we produced CRISPR/Cas9 CTSB knockout clones, which were utilized to evaluate treatment sensitivity to three different ASNases: two wildtype enzymes, one from *E. coli* (EcA_WT) and other from *E. chrysanthemi* (ErA_WT), and a double-mutant enzyme from *E. chrysanthemi* (ErA_DM) that is more active and presents a lower glutaminase/asparaginase activity ratio [16]. In all conditions, CTSB KO cells remained resistant to ASNase treatment, as demonstrated by the retention of ALL cell viability and IC_50_, implicating additional key factors, such as tumor environment and immune system, in the inherent or induced ASNase resistance and, ultimately, inefficient cancer treatment.

## 2. Results

### 2.1. Production of Cathepsin B Knockout Clones in REH ALL Cells

To investigate the role of CTSB on ASNase treatment resistance, we generated *CTSB* KO cells utilizing the clustered regularly interspaced repeats with associated protein 9 (CRISPR/Cas9) system. Five different RNA guides (sgRNAs), targeting different regions of the *CTSB* gene, were designed and cloned into LGR plasmid. Viral particles were produced by transfecting HEK293T cells with CTSB targeting constructs (LGR + sgRNA-1–5) or with control sgRNA constructs LGR + sgRNA-Rosa26 (non-targeting, negative control of target edition) and LGR + sgRNA-RPA3 (essential human gene, positive control of system edition).

According to expression data analyzed in the Human Protein Atlas, the Hep-G2 cell line presents tenfold higher normalized transcript per million (nTPM) of *CTSB* mRNA than the REH cell line (https://www.proteinatlas.org/ENSG00000164733-CTSB/cell+line, accessed on 28 May 2023). Thus, lentiviral particles were first used to infect Hep-G2 cells, allowing the prediction of sgRNAs with *CTSB* targeting efficiency. Flow cytometry analysis of sgRNA-expressing Hep-G2 cells 15 days post infection demonstrated 70–80% GFP-expressing cells (Figure S1A) in conditions bearing *CTSB* sgRNAs and those of the *Rosa26* non-targeting control. As expected, this contrasted with cells infected with sgRNA targeting the essential gene *RPA3*, which were depleted of GFP^+^ cells (Figure S1B). To verify the efficiency and specificity of sgRNAs in generating CTSB KO cells, we evaluated protein expression by Western blot. Three out of the five clones (sgRNAs #1, #3, and #4) expressed less CTSB protein than cells expressing *Cas9* alone or *Rosa26* non-targeting control, confirming the efficiency of *CTSB* targeting with sgRNAs #1, #3, and #4 (Figure S1C).

**Figure 1:**
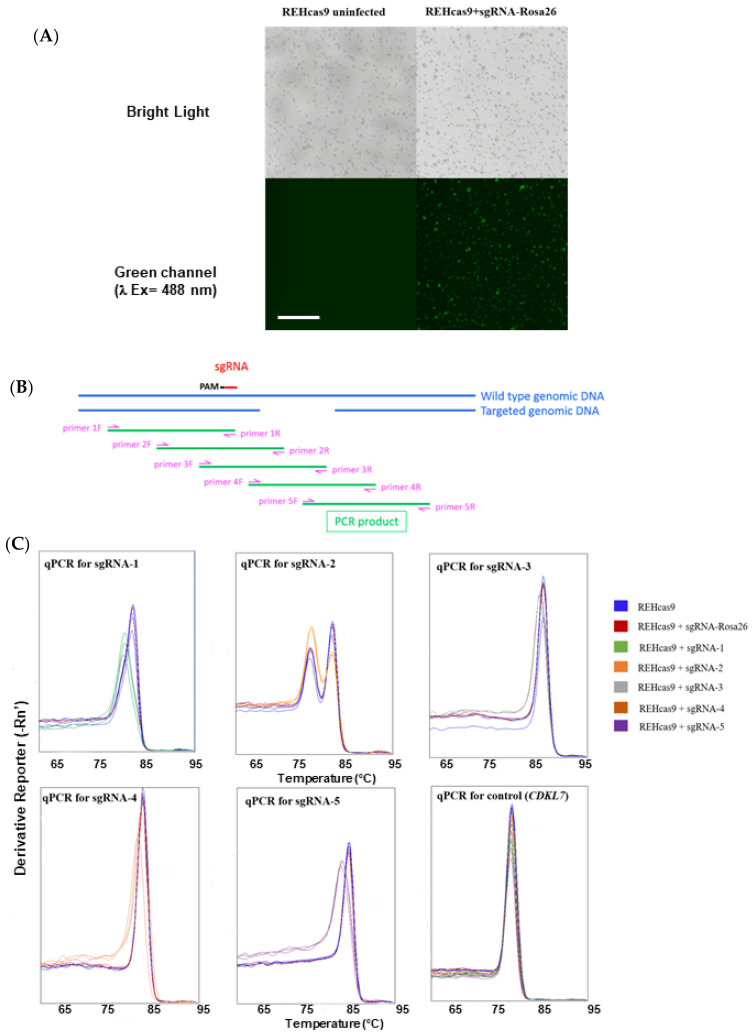
Monitoring of REHcas9 cell infection and CTSB edition. (**A**) Representative image of REHcas9 infection using lentivirus particles sgRNA-Rosa26. The same procedure was carried out for all lentiviruses produced (sgRNA-1–5 for *CTSB* gene and sgRNA-RPA3). The infection was confirmed by GFP expression in inverted fluorescence microscope. The figure shows the uninfected cells and infected cells with sgRNA-Rosa26 lentivirus expressing GFP under bright light and green channel excitation at 488 nm. Scale bar, 100 µm. (**B**) Schematic representation of gene and qPCR-based assay to estimate gene editing efficiency using CRISPR/Cas9 editing tool. Five different primers pairs were designed to flank the target region for the knockout of each sgRNA to validate the editing of the *CTSB* gene by qPCR. (**C**) Confirmation of *CTSB* gene editing by qPCR. Derivate melting curves from qPCR of REHcas9 cells infected with different sgRNAs. The genomic DNA of each cell was used for *CTSB* editing validation. Uninfected REHcas9 cells and REHcas9 cells infected with sgRNA-Rosa26 (REHcas9 + sgRNA-Rosa26) were used as a negative control (no editing in *CTSB* gene). The specific primer for the cyclin-dependent kinase 7 (*CDKL7*) gene was used as an internal control.

Next, we analyzed the effects of *CTSB* targeting REH cells, using the same approach as described for the generation of CTSB KO Hep-G2 cells (Figure 1A). To confirm *CTSB* gene editing in REHcas9 cells, genomic DNA from GFP-expressing cells was extracted and analyzed by real-time quantitative polymerase chain reaction (RTqPCR), with primers flanking the target regions of the sgRNAs on the *CTSB* gene (Figure 1B). Analysis of the dissociation curves of REHcas9 targeted with different *CTSB* sgRNAs indicated a distinct dissociation peak from those present in uninfected REHcas9 cells, thus suggesting alteration of the nucleotide composition of the *CTSB* gene in cells targeted with CRISPR/Cas9, apart from the sgRNA-2 that showed multiple PCR products. Therefore, the editing of *CTSB* for sgRNA-2 was inconclusive (Figure 1C).

**Figure 2:**
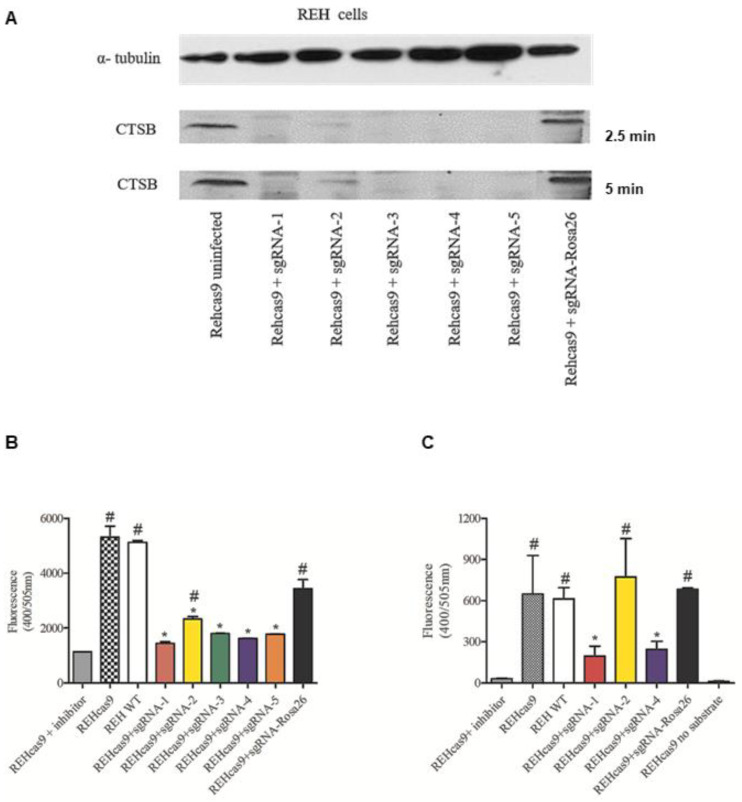
Evaluation of CTSB protein amount and activity in REHcas9 + sgRNAs. (**A**) Western blotting assay image using REHcas9 + sgRNAs. Uninfected REHcas9 and REHcas9 + sgRNAs cells were lysed, 150 µg of total protein was used in each sample, and the film was exposed for 2.5 and 5 min. α-Tubulin protein (60 kDa) was used as a total protein load control. (**B**) Cathepsin B activity assay of uninfected (in the presence or absence of CTSB inhibitor) and infected cells with sgRNAs (1–5) and Rosa26 using the Cathepsin B Activity Fluorometric Assay Kit (BioVision, Waltham, MA, USA). (**C**) CTSB activity assay using Sigma-Aldrich reagents (see Section 4) for analysis of lysates of REHcas9 cells and cells infected with sgRNAs-1, 2, and 4 and Rosa26. Results represent the mean ± standard deviation of experiments performed in triplicate. One-way ANOVA statistical analysis followed by Tukey’s post hoc test showed a significant difference (*p* < 0.05) between the CTSB activities of sgRNA-infected cells (1–5) when compared to the uninfected control REHcas9 cells (*) or when compared to cells treated with CTSB inhibitor (#).

The results were also confirmed with the analysis of protein levels, which indicated that all conditions expressing sgRNAs were depleted of CTSB protein (Figure 2A). Next, CTSB activity in CTSB KO cell lines was quantified by two fluorescence-based assays (Figure 2B,C). Here, equivalent numbers of cells (REHcas9 + sgRNAs) were used in the reaction. We also evaluated the enzymatic activity in REH WT cells, without the presence of Cas9 protein, which showed the same CTSB activity as the uninfected REHcas9 cells. Across all KO conditions, our analysis showed a statistically significant reduction in activity when compared to uninfected cells, with the greatest activity reductions observed on clones #1 and #4, thus confirming the CTSB-deficient state of these cells (Figure 2B). Further enzymatic activity analysis comparisons across conditions confirmed the significant reduction in the activity of sgRNA-1 and sgRNA-4 clones in relation to controls, thus supporting their truly KO phenotype (Figure 2C).

**Figure 3:**
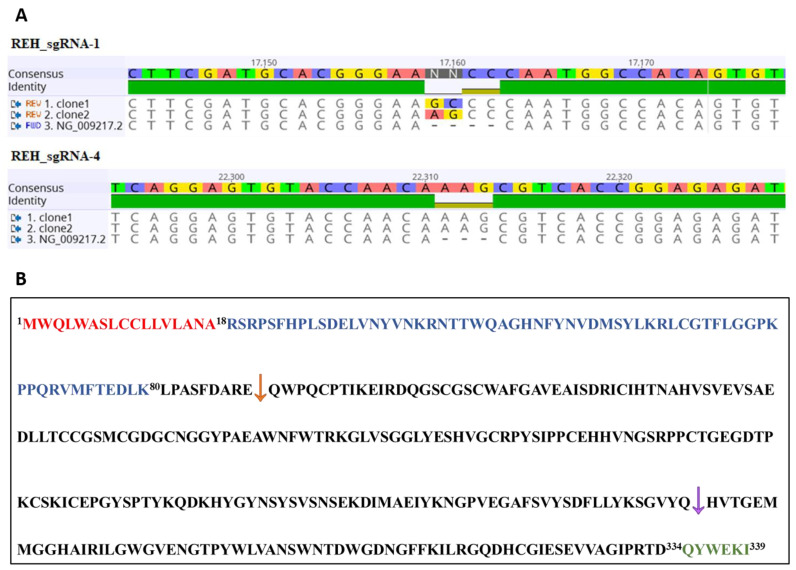
Analysis of *CTSB* gene editing in confirmed REHcas9 cells with *CTSB* knockout by sequencing. (**A**) Analysis of gene editing by sequencing of REHcas9 + sgRNA-1 and REHcas9 + sgRNA-4. The plasmids obtained from TOPO + PCR constructs were sequenced using the TOPO M13 vector primer and aligned with the *CTSB* gene sequence (NG_009217.2) using Geneious^®^ 11.1.5 software. (**B**) Human CTSB protein sequence (Uniprot code: P07858). The signal peptide sequence is identified in red; the propeptide sequence is identified in blue; the mature protein sequence is identified in black; the C-terminal propeptide sequence is identified in green. The arrows represent the position at the beginning of the changes in the amino-acid sequence caused by the editing of sgRNA-1 (orange arrow) and sgRNA-4 (purple arrow).

Additional DNA analysis indicated the specific insertions of bases that disrupted the expression/function of *CTSB* gene (Figure 3A). For sgRNA-1, the insertion of four bases resulted not only in the alteration of the amino acid from residue 89, but also in the transcription of a translation stop codon (TGA) at position 120, generating a truncated CTSB (Figure 3B). For cells expressing sgRNA-4, the insertion of AAG bases in the middle of the CAC codon resulted in the alteration of two amino acids in the protein sequence (Table 1). Both editions provided mutations in the mature protein (Figure 3B).

**Figure 4:**
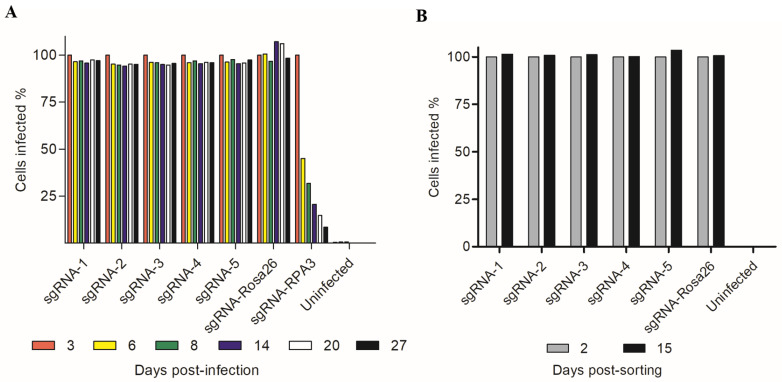
Monitoring of REHcas9-sgRNA cell proliferation by flow cytometry. (**A**) Cells infected with different sgRNAs were monitored by flow cytometer MACSQuant^®^ (Miltenyi Biotec) for 27 days. (**B**) After 27 days, cells were sorted by fluorescence-activated cell sorting (FACS) and monitored for additional 15 days.

After obtaining CTSB KO REH cell lines, we investigated the effects of CTSB deletion on REH cell normal growth. In doing so, REHcas9 cells were monitored for 27 days via quantification of GFP levels across all conditions (Figure 4A). With this approach, we established that depletion of CTSB did not impact cell survival, given that the GFP levels of REH cells bearing *CTSB* sgRNAs, and those from the non-targeting control *Rosa 26* condition remained steady through the analysis. These results were supported by the GFP level monitoring analysis of FACS-isolated CTSB KO cells and Rosa26-targeted cells, which remained unchanged over time (Figure 4B). This was in marked contrast to cells expressing sgRNA targeting the essential gene *RPA3*, which progressively lost cells expressing GFP, indicating the negative selection of *RPA3*-targeted cells (Figure 4A). These results support that REH ALL cells do not depend on the expression of CTSB for overall survival and proliferation.

### 2.2. Evaluation of the Viability of REHcas9 + sgRNA-1 Cells after Treatment with ASNase

**Figure 5:**
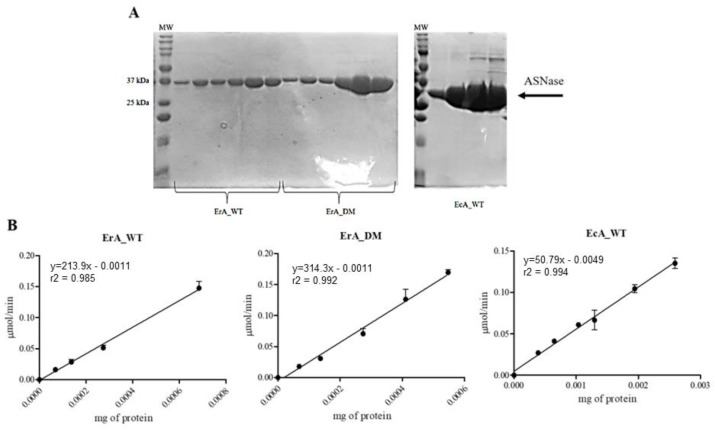
Purification and activity assay of ASNases. (**A**) Evaluation of purity of ErA_WT, ErA_DM, and EcA_WT proteoforms by 12% SDS-PAGE gel electrophoresis. ErA_WT and ErA_DM proteins have ~37 kDa and the EcA_WT protein has ~35 kDa. MW indicates the molecular weight Dual Color Standards ^TM^ (Bio-Rad). (**B**) Specific activity of ErA_WT, ErA_DM, and EcA_ WT proteoforms measured by Nessler’s reagent. Graph of reaction speed in μmol/min as a function of the amount of protein in milligram. The slope of the line equation represents the specific activity for each enzyme given in U/mg. One unit (U) is equal to 1 µmol of ammonium produced per minute at 37 °C. The points represent the mean ± standard error (n = 3).

Using the REHcas9 + sgRNA-1 described above, we performed the cytotoxicity assay in the presence of three different ASNases (EcA_WT, ErA_WT, and ErA_DM). EcA_WT and ErA_WT are the enzymes already commercially used for the treatment of ALL. ErA_DM is a double-mutant enzyme obtained from the ErA_WT enzyme, developed by our group, which has different kinetic and immunological properties [16]. These enzymes were produced by heterologous expression in *E. coli* BL21(DE3). After expression, selection of pure fractions was performed by analysis of SDS-PAGE pattern (Figure 5A). High-purity samples were used to determine the specific activity for L-asparagine (Figure 5B) and then used in the cytotoxicity assay.

**Figure 6:**
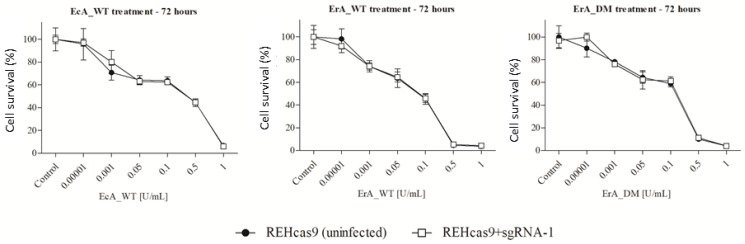
In vitro cytotoxicity assay of CTSB KO cells in the presence of ASNase. REHcas9 (uninfected) and REHcas + sgRNA-1 cells were treated with different concentrations of EcA_WT, ErA_WT, and ErA_DM enzymes for 72 h. Cells without treatment and with the addition of the buffer in which the enzymes were diluted are named “control”. The assay was performed in analytical triplicate, and the data represent the mean ± standard deviation (n = 3).

REHcas9 and REHcas9 + sgRNA-1 cells were treated with different concentrations of ASNases for 72 h, and then cell viability was determined. Unexpectedly, in all conditions, no significant differences were found in the IC_50_ values (Table 2). Furthermore, in all the conditions tested, the cell viability profile was the same between REHcas9 and REHcas9 + sgRNA1 cells (Figure 6).

## 3. Discussion

There are different ALL cells with different phenotypes and alterations in gene expression, such as in the expression of proteases. For example, the REH ALL lineage is capable of constitutively expressing *CTSB* [4], as well as high levels of asparagine synthetase (*ASNS*), unlike other ALL cell lines [15]. It is known that ASNS and CTSB proteins are directly related to the ALL patient response to ASNase treatment [4,5,6,17].

The REH ALL cell line is a pre-B cell (BCP-ALL) and has the *ETV6/RUNX1* translocation. The *ETV6* gene encodes the *ETS* family of transcription factors (E26 transformation specific), responsible for the differentiation and growth of hematopoietic cells, and the *RUNX1* gene encodes the transcription factor responsible for the expression of genes involved in the stem-cell differentiation process [18].

BCP-ALL is the most common cell type in ALL cases [14], and the *ETV6/RUNX1* phenotype represents 30% of B-ALL cases [19]. It has been shown that patients with the *ETV6/RUNX1* phenotype who experience severe adverse effects and require discontinuation of ASNase have a worse prognosis in terms of disease-free survival [20].

*CTSB* expression levels were evaluated in BCP-ALL cells obtained from ALL patients. Cells were classified into subtypes according to the mutations found; however, the group of patients identified with the *ETV6/RUNX1* translocation (the phenotype characteristic of REH cells) was small. Analysis of gene expression in this group suggests a high expression of CTSB, although the small number of patients with this translocation was a limitation of the research extrapolation [21]. The level of *CTSB* expression in REH cells is still unclear, but the work of Wrona (2019) suggested a high expression of this gene in primary cultured cells. In addition, there was evidence of CTSB production by REH cells in previous studies [4]. We found a low expression level of CTSB protein in our experimental conditions.

Patel et al. (2009) [4] showed that REH cells do not express AEP, but express CTSB. Accordingly, CTSB inhibitor (CA074Me—CTSBi) completely abolished ASNase degradation by this cell lysate, while, as expected, AEP inhibitor (MV026630—AEPi) had no effect. To extrapolate these data to ALL patients, primary blast cells lysates were also tested; two samples out of four presented the same behavior as the REH cell lysate, while the other two samples required both inhibitors (CTSBi + AEPi) to prevent ASNase degradation, suggesting a role of these proteases in leukemia cell ASNase resistance [4].

We know that the expression/regulation of the CTSB passes through several complex pathways, ranging from transcription to post-transcriptional modification, translation, glycosylation, maturation, and modulation, which can be regulated by zymogen activation, pH, or endogenous inhibitors. The enzyme can be found in lysosomes, the cytoplasm, and the plasma membrane, on the pericellular surface, or even secreted into the extracellular medium [9]. Given the complexity of its regulation, the expression of CTSB can occur in different ways in vivo and in vitro.

We obtained a high infection rate and confirmed the gene editing by means of RTqPCR, followed by Western blotting and enzymatic activity. Editing efficiency was also observed in Hep-G2 cells, which express high levels of CTSB, showing our experimental approach accuracy. When we sequenced the genomic DNA of edited REHcas9 cells, we observed the presence of a frameshift mutation in the cells edited by sgRNA-1 and the insertion of two amino acids in the cells edited by sgRNA-4. In REHcas9 + sgRNA-1 cells, the presence of an early translation stop codon justified the absence of the protein in Western blotting and the significant reduction in CTSB activity in these cells, equivalent to cells treated with a CTSB inhibitor. In REHcas9 + sgRNA-4 cells, the same effect was observed with the insertion of the amino acids Gln and Ser in the protein.

However, analyzing the cellular viability of the REHcas9 + sgRNA-1 after treatment with the different ASNases, we concluded that both the IC_50_ values and the toxicity profile showed no differences when compared to the control. 

Even though the CTSB protein is constitutively expressed by REH cells, and even though their lysates present active CTSB able to degrade ASNase in vitro (4), the conditions tested here show that the level of CTSB in intact REH cells does not influence the response to ASNase treatment. Although the REH ALL cells are considered resistant to treatment, other conditions have also been associated with ASNase treatment resistance such as the expression of asparagine synthetase in *ETV6–RUNX1* cell lines, which may be related to an inferior outcome for patients. A study about the association between genetic subtypes of BCP-ALL and the expression of ASNS showed that the relative mRNA expression of *ASNS* was the highest in the *ETV6–RUNX1* fusion group [21]. Moreover, therapeutic resistance is often impacted by the patient immune system, either by macrophage-driven ASNase clearance, inactivation of the enzyme by neutralizing antibodies, or the accumulation of less cytotoxic NK cells in patients with B/T-ALL [2,5,17,22]. Therefore, multiple factors can be strongly associated with L-asparaginase use effectiveness and with the patient survival. In conclusion, our group previously related ASNase resistance to CTSB with immunological profile and serum half-life in vivo (testing health mice model) [2]; however, here, we observed that the expression and activity of CTSB in ALL cells are not directly related to resistance to ASNase, at least in the REH cell line. The resistance to ASNase seems to be highly influenced by immune cell recognition and systemic response rather than the secretion or membrane surface activity of proteases within ALL cells.

## 4. Materials and Methods

### 4.1. sgRNA Oligo Design and Cloning in the LGR Plasmid

For the construction of the CRISPR/Cas9 system and design of the sgRNAs for *CTSB* editing, we used the sequence of the coding gene for human Cathepsin B ID 1508. The oligonucleotides for the sgRNA were designed using the Geneart^®^ CRISPR Search and Design Tool (Thermo Fisher Scientific, Waltham, MA, USA) (https://apps.thermofisher.com/apps/crispr/index.html#/search, accessed on 10 January 2020), and five of them, with a score above 70 to avoid off-target effects, were chosen (Table 3).

For the cloning of sgRNA in LGR plasmid (Lenti_sgRNA-EFS_GFP), the methodology previously described by Shi et al. [23] was followed. All sgRNA oligos (Table 3) were previously phosphorylated before cloning into the LGR vector using the T4 PNK enzyme (New England Biolabs^®^ Inc., Ipswich, MA, USA). In the reaction, we added 2.5 µM of forward oligo, 2.5 µM of reverse oligo, 1× T4 binding buffer, and 5 U of T4 PNK enzyme, in a final volume of 10 µL. The reaction was incubated at 37 °C for 30 min, at 95 °C for 5 min, and then ramped down to 25 °C at 5 °C/min.

Next, 5–10 μg of LGR plasmid (Addgene plasmid # 65656, Watertown, MA, USA) [23] was digested with 2 U of *Bsmb1* restriction enzyme (New England Biolabs^®^ Inc.), with 1× NEBbuffer 3.1 in a final volume of 30 μL. The reaction was incubated for 2 h at 55 °C before immediately adding 7 U of calf intestinal alkaline phosphatase enzyme (CIP) (New England Biolabs^®^ Inc.) at 37 °C for 10 min, in order to prevent vector recircularization. The reaction was inactivated at 80 °C for 20 min. The linearized vector was visualized by DNA electrophoresis in 1% agarose gel in TAE 1× buffer with the addition of 1.5 μg/mL ethidium bromide. The linearized LGR plasmid was extracted and purified with QIAquick^®^ Gel Extraction kit (Qiagen, Hilden, Germany) following the manufacturer’s protocol, and the DNA concentration was quantified using NanoDrop One (Thermo Fisher Scientific).

The sgRNA phosphorylated oligos were previously diluted in 10 mM Tris pH 8.0 in the ratio of 1:200. The reaction was composed of 4 μL of sgRNA oligos (1:200), 1 μL of LGR digested, 1 μL of 10× T4 DNA ligation buffer, and 1 μL of T4 DNA ligase enzyme (New England Biolabs^®^ Inc.) in a final volume of 10 μL; then, it was incubated for 30 min at room temperature. The ligation mix was used to transform One Shot^TM^ Stbl3^TM^ chemically competent *E. coli* strain (Invitrogen–Thermo Fisher, Waltham, MA, USA). 

To confirm the correct construction of LGR + sgRNA for *CTSB* edition, the transforming clones were isolated and grown in 5 mL of LB + 100 μg/mL of ampicillin at 37 °C overnight, shaking at 250 rpm. Thereafter, the plasmid was extracted using the QIAGEN Plasmid Mini Kit (cat. #12123), as described by the manufacturer. The DNAs were quantified using NanoDrop One (Thermo Fisher Scientific), 1.0 μg of each construction, and 5 μM of U6 promoter primer (5′–AACCGGTGCCTAGAGAAGGT–3′) were sent for sequencing at Cold Spring Harbor Laboratory’s (Cold Spring Harbor, NY, USA) DNA Sequencing Facility.

### 4.2. Cell Culture

All cells used in this study are periodically authenticated by CSHL (USA). Before use, all of them were verified for the absence of *Mycoplasma* through PCR. 

Human wildtype (WT) cells were obtained from the American Type Culture Collection (ATCC) (https://www.atcc.org/, Manassas, VA, USA). Cells expressing cas9 protein were previously produced from WT cells by Professor Dr. Christopher Vakoc of CSHL and kindly provided for this study.

HEK293T cells were cultured in DMEM + 5% FBS + 1% penicillin/streptomycin (P/S) (Gibco, Grand Island, NY, USA). Hep-G2cas9 cells were cultured in MEM + 5% FBS + 1% P/S. REH WT and REHcas9 cells were cultured in RPMI 1640 GlutaMAX^TM^ + 5% FBS + 1% P/S. The cells were incubated at 37 °C under a 5% CO_2_ atmosphere, and the culture medium was changed every 48 or 72 h. 

For the suspension cells REH, the cultures were centrifuged at 300× *g* for 3 min, the supernatant was removed, and fresh culture medium was added.

For the adhered cells HEK293T and Hep-G2, the culture medium was removed by aspiration, and fresh medium was added immediately. When expansion and/or counting was necessary, the cells were washed with 1× phosphate-buffered saline (PBS) (137 mM NaCl, 2.7 mM KCl, 10 mM Na_2_HPO_4_, and 1.8 mM KH_2_PO_4_), and then a solution of trypsin TripLE^®^ Express (Gibco) was added to dissociate the adhered cells. After, cells were centrifuged at 300× *g* for 5 min, the supernatant was removed, and fresh culture medium was added.

Cell counting was performed by adding Trypan Blue solution (Sigma-Aldrich, San Luis, MO, USA) in cell samples that were analyzed by the TC20^TM^ Automatic Cell Counter (Bio-Rad, Hercules, CA, USA).

### 4.3. Virus Particles Production

The constructed plasmids were used to generate lentivirus by transfecting HEK293T cells using 3 µg of LGR + sgRNA, 2 µg of pPAX2 (a gift from Didier Trono-Addgene plasmid # 12260), and 0.8 µg of pMD2.G (a gift from Didier Trono-Addgene plasmid # 12259) mixed in 200 µL of Opit-MEM^TM^ + 15 µL of polyethylenimine (PEI) (Polyscience, Warrington Township, Bucks County, PA, USA). 

One day before transfection, approximately 7 × 10 ^5^ HEK 293T cells per well were harvested; on the transfection day, the medium was removed, and the mixture of plasmids (described above) for lentivirus production was added to cells with 1 mL of DMEM + 5% FBS before incubating at 37 °C under 5% CO_2_. After 6 h, the medium was removed, and 1 mL of fresh DMEM medium + 5% FBS + 10 mM HEPES + 1% P/S was added before incubating overnight; then, the culture medium was changed again. After 24 h, the medium was collected (containing the viral particles), and 1 mL of DMEM + 5% FBS + 10 mM HEPES + 1% P/S was added to adhered cells. Viral particles were collected every 24 h for 3 days.

### 4.4. Cell Infection

Approximately 1 × 10^5^ cells (REHcas9 or Hep-G2cas9) per well were seeded in a 12-well plate in 200 µL of appropriate culture medium (RPMI or MEM) before adding 1 mL of lentivirus produced, as described above. The plate was centrifuged at 1000× *g* for 25 min at room temperature. For REHcas9 cells, after the centrifugation step, the cells were gently resuspended. The cells were incubated at 37 °C under 5% CO_2_ atmosphere for 24 h, and then 1 mL of fresh medium was added. After 24 h, the culture medium was removed, fresh medium was added, and the cells were incubated at 37 °C under 5% CO_2_ atmosphere for another 24 h. For REHcas9 cells, the cell culture was centrifugated at 300× *g* for 3 min to remove the medium. On the third day of infection, GFP expression was checked under an inverted fluorescence microscope Nikon Eclipse Ti (Nikon, Tokyo, Japan) using the green channel at an excitation wavelength of 488 nm; the infection rate was monitored by flow cytometry using an MACSQuant^®^ Analyzer 10 instrument (Miltenyi Biotec, Bergisch Gladbach, Germany).

### 4.5. Genomic DNA Extraction and qPCR

The cells were resuspended in three volumes of cold 1× PBS and centrifuged at 500× *g* at room temperature. Supernatant was removed, the pellet was added to three volumes of lysis buffer (50 mM Tris pH 8.0, 10 mM EDTA, and 10% sodium dodecyl sulfate (SDS)), and the tube was inverted several times. Then, 5 µL of RNase A (10 mg/mL) was added, before incubating for 30 min at 65 °C; then, 3 µL of Proteinase K (20 mg/mL) was added, before incubating for 20 min at 40 °C. After incubation, the volume was completed to approximately 400 µL with TE buffer (10 mM Tris-HCl pH 8.0 and 1 mM EDTA), 400 µL of buffered phenol was added, and the mixture vortexed for 1 min. The sample was transferred to a pre-spin lock phase and centrifuged at 12,000× *g* for 5 min. The upper phase was collected, before adding 10% volume of sodium acetate 3 M and three volumes of cold ethanol 100%. After centrifugation at 12,000× *g* for 5 min, the ethanol was removed, and the precipitated DNA was washed with 700 µL of cold ethanol 70%. The sample was centrifuged, and the ethanol was removed. The DNA pellet was resuspended in 50 µL of 10 mM Tris pH 8.0 and quantified using NanoDrop One (Thermo Fisher Scientific, USA).

Primers were designed covering the target region for the knockout of each sgRNA (Table 4), to validate the edition of the *CTSB* gene by qPCR. In the qPCR reaction, we used 200 ng of the genomic DNA, 0.7 µM of the forward and reverse primers, and 5 µL of SYBR Green PCR Master Mix (Biosystem, Barcelona, Spain), for a final volume of 10 µL, in a thermocycler QuantStudio 12K Flex Real-Time PCR System (Thermo Fisher Scientific, USA).

### 4.6. TOPO Cloning for Confirmation of CTSB Editing by Sequencing

Genomic DNA extracted from REHcas9 + sgRNA cells was used as a template to amplify *CTSB* gene regions using the primers described in Table 4 and cloned in the TOPO vector, using TOPO^®^TA Cloning Kit (Invitrogen), following the manufacturer’s instructions. The clones were selected in medium containing 100 µg/mL of ampicillin and 80 µg/mL of X-gal (5-bromo-4-chloro-3-indolyl β-d-galactopyranoside), and then three isolated white colonies were cultured in LB medium for subsequent plasmid extraction using the QIAGEN Plasmid Mini Kit (cat. #12123) as described by the manufacturer. Plasmids were quantified by NanoDrop One (Thermo Fisher Scientific, USA), and 1.0 μg of each construction and 5 μM of M13 primers (forward 5′–GTAAAACGACGGCCAG–3′ and reverse 5′–CAGGAAACAGCTATGAC–3) were sent for sequencing at Cold Spring Harbor Laboratory’s DNA Sequencing Facility.

### 4.7. Western Blotting

The cells REH or Hep-G2 were centrifuged at 500× *g* for 5 min and resuspended with NP-40 lysis buffer (50 mM Tris-HCl pH 8.0, 150 mM NaCl, 1% NP-40, and 5 mM EDTA), and then kept at 4 °C under agitation for 30 min, followed by centrifugation at 15,000× *g* for 20 min at 4 °C (adapted from [4,5]). The supernatant was recovered, and the quantity of total protein was measured using Bradford’s reagent (Bio-Rad); the standard curve was made with known concentrations of bovine serum albumin (BSA). When necessary, the protein extract was concentrated using Amicon^®^ Ultra 3K centrifuge filters (Merck Millipore, Burlington, MA, USA) at 7500× *g* for the time necessary to apply the desired amount of protein into the gel. For samples obtained from REH cells, up to 150 µg of total protein was applied to gel electrophoresis, while, for samples obtained from the Hep-G2 cells, up to 90 µg of total protein was used.

Electrophoresis was performed as described by Laemmli (1970). Transfer was performed using the iBlot^TM^ Transfer Stack kit in a 0.4 µm PVDF membrane (Invitrogen) with the iBlot^TM^ Dry Blotting System (Invitrogen) for 2 min.

After transfer, the membranes were washed with 1× TBS-T (2.4 g of Tris-Base, 8.8 g of NaCl, and 1 mL of Tween-20 in 1 L of H_2_O) three times for 10 min, blocked with 5% of nonfat dried milk diluted in 1× TBS-T for 1 h at room temperature, and washed once with 1× TBS-T. The primary anti-cathepsin B antibody (Sigma-Aldrich, #C6243) and anti-α-tubulin antibody (Millipore-#CP06, Burlington, MA, USA), both from mouse, were added, and membranes were incubated overnight at 4 °C. Subsequently, the membranes were washed again with 1× TBS-T and incubated with anti-mouse HRP-conjugated antibody for 1 h. The HRP signal was developed with Immobilon Crescendo Western HRP substrate (Millipore) in X-ray CL-XPosure Film (Thermo Fisher Scientific, USA). 

### 4.8. Cathepsin B Activity Assay

The cells were counted using Trypan Blue (Sigma Aldrich Co., Saint Louis, MO, USA), and 10^6^ cells were used for each replicate. 

In the first assay, CTSB enzyme activity was measured using the CTSB Activity Fluorometric Assay Kit (BioVision, Waltham, MA, USA). The activity assay was carried following the manufacturer’s instructions. The reaction was incubated at 37 °C for 90 min, and then the fluorescence was measured with 400 nm excitation and 505 nm emission filters in a EnVision Multimode Plate Reader spectrophotometer (PerkinElmer, Waltham, MA, USA).

In the second assay, cells were centrifuged at 500× *g* for 5 min, washed with 1× PBS, and centrifuged again. The supernatant was discarded, and the cells were resuspended in 100 µL of Bug Buster Protein Extraction Reagent (Novagen-Merck Millipore, Burlington, MA, USA), kept under gentle agitation for 20 min, and centrifuged at 16,000× *g* for 10 min; the supernatant fraction was recovered. In the assay, the fluorescent substrate for CTSB Z-Arg-Arg-7-amido-4-methylcoumarin hydrochloride (Z-RR-AMC-Sigma-Aldrich #C5429) was used for the reaction, and the inhibitor for CTSB CA-074 Methyl-ester (Sigma-Aldrich #C5857) was used as a control. The reaction consisted of 10 µL of 10× assay buffer (500 mM sodium acetate pH 5.2, 50 mM EDTA, and 50 mM DTT), 33 µL of sample (cell lysis supernatant described above), 20 µM of substrate Z -RR-AMC, and water to a final volume of 100 µL. For the blank, the cell extract was not added to the reaction; as a control, the reaction was carried out without substrate. For the negative control, 10 µM of inhibitor CA-074 methyl-ester was added to the reaction. The reaction was incubated at 37 °C for 90 min, and then the fluorescence was measured with 400 nm excitation and 505 nm emission filters in a spectrofluorometer (Spectra Max M2–Molecular Devices, San José, CA, USA) [24].

### 4.9. Obtaining the Enzymes L-Asparaginases for Cell Viability Assay

Wildtype ASNase from *E. chrysanthemi* (ErA-WT) and double-mutant ASNase from *E. chrysanthemi* (ErA-DM) were expressed in *E. coli* BL21(DE3) in LB medium using 0.1 mM of IPTG for induction for 22 h. After that, the pellet was subjected to osmotic shock to obtain periplasmic proteins and then purified by cation-exchange chromatography [16]. 

Wildtype ASNase from *E. coli* (EcA-WT) was expressed in *E. coli* BL21(DE3) in LB medium using 1 mM of IPTG for induction for 22 h. After that, the pellet was subjected to osmotic shock to obtain periplasmic proteins, and then purified by anion-exchange chromatography, followed by size exclusion chromatography [2].

Purification was confirmed by 12% SDS-PAGE reducing gel electrophoresis [25].

The ASNase specific activity assay was performed in 96-well microplates using 50 mM potassium phosphate pH 8.0 for ErA-WT and ErA-DM or 50 mM Tris-HCl pH 8.8 for EcA-WT and 20 mM L-asparagine (Sigma-Aldrich). The enzyme concentration varied from 5 to 50 nM for ErA-WT and ErA-DM [16] and from 30 to 200 nM for EcA_WT enzyme [2]. The reaction was incubated at 37 °C for 10 min, and then the reaction was stopped with 20 µL of trichloroacetic acid 1.5 M and diluted 10× in water; finally, 37 µL of Nessler’s reagent (Sigma) was added. The reading was performed at 440 nm in a spectrophotometer En Vision Multimode Plate Reader (PerkinElmer). The standard curve was made with ammonium sulfate under the same conditions as the tests. The absorbance values obtained were interpolated in this standard curve.

### 4.10. Cell Viability/Cytotoxicity Assay

A total of 2 × 10^4^ cells were seeded in 150 µL of culture medium in each well with different concentrations of ASNases (from 0.01 to 1 U/mL—indicated in the figures); plates were incubated at 37 °C under 5% CO_2_ for 72 h. After incubation, 0.5 mg/mL 3-(4,5-dimethyl-2-thiazolyl)-2,5-diphenyl-2H-tetrazolium bromide (MTT) diluted in 1× PBS was added, and the plate was incubated at 37 °C under 5% CO_2_ for 3 h. To solubilize the formed crystals, 150 µL of a 10 mM solution of hydrochloric acid + 10% SDS was added and incubated overnight at 37 °C under 5% CO_2_. Then, the absorbance was measured at 570 nm in a spectrophotometer (Spectra Max M2–Molecular Devices).

## Figures and Tables

**Table 1:** Analysis of edits in the DNA and protein sequence.

	DNA			Protein		
	Edit Position *	Inserted Bases	Frame Change	AAs Change	First AA Change	Stop Codon
sgRNA-1	265	GGCC or AGCC	Yes	89 to 120	SER or ALA	120
sgRNA-4	807	AAG	No	269 and 270	GLN and SER	340

* The position of the nitrogenous base was identified considering the sequence used in the transcription.

**Table 2:** IC_50_ values (U/mL) obtained by the MTT cytotoxicity assay.

	EcA_WT	ErA_WT	ErA_DM
REHcas9	0.11	0.080	0.10
sgRNA-1 *	0.14	0.082	0.10

* Statistical analysis: two-way ANOVA followed by Bonferroni post hoc test (*p* < 0.05) when compared with REHcas9 cells within the same treatment.

**Table 3:** Primers designed encoding sgRNA for *CTSB* gene.

Oligo	Forward 5′ → 3′	Reverse 5′ → 3′
**sgRNA-1**	**CACC**GGCTTCGATGCACGGGAACAA	**AAAC**TTGTTCCCGTGCATCGAAGC
**sgRNA-2**	**CACC**GTTCCACGCACCGATCAGTAC	**AAAC**GTACTGATCGGTGCGTGGAA
**sgRNA-3**	**CACC**GCACCAATGCGCACGTCAGCG	**AAAC**CGCTGACGTGCGCATTGGTG
**sgRNA-4**	**CACC**GTCATCTCTCCGGTGACGTGT	**AAAC**ACACGTCACCGGAGAGATGA
**sgRNA-5**	**CACC**GGCCGTTGACGTGGTGCTCAC	**AAAC**GTGAGCACCACGTCAACGGC

In bold are highlighted the sticky ends for the insertion into digested plasmid with the *Bsmb1* restriction enzyme (New England Biolabs^®^ Inc., Ipswich, MA, USA).

**Table 4:** Primers to qPCR: sequences flank the editing regions of CTSB gene.

Construction	Forward 5′ → 3′	Reverse 5′ → 3′
LGR + sgRNA-1	CCTGGTCTCTGATCTCTTTGATG	TGTGGGATCAGAGCTTGTAATG
LGR + sgRNA-2	CAGACCCTGTCTGAAACTTGTA	TTCCACGCACCGATCAG
LGR + sgRNA-3	CAGGGTCTCTCAGCACTAAAC	GGATCTGCATCCACACCAA
LGR + sgRNA-4	CAGTAGGGTGTGCCATTCTC	CTCAGCCAGTTCTTCCCTTTT
LGR + sgRNA-5	TCACAGATCTTGCTACACTTG	GTAGGTTGACTCCGCTTTCTC

## Data Availability

All data are contained within the manuscript.

## Supplementary Materials

The following supporting information can be downloaded at: https://www.mdpi.com/article/10.3390/ijms241311215/s1.
