# Supplementary material for: Cathepsin B Is Not an Intrinsic Factor Related to Asparaginase Resistance of the Acute Lymphoblastic Leukemia REH Cell Line
Source: Int J Mol Sci. 2023 Jul 7;24(13):11215. doi: 10.3390/ijms241311215 (PMC10342508; doi:10.3390/ijms241311215)
Supplement: Supplementary file 1 [file ijms-24-11215-s001.zip › ijms-2451804-supplementary.pdf]

Supplementary material

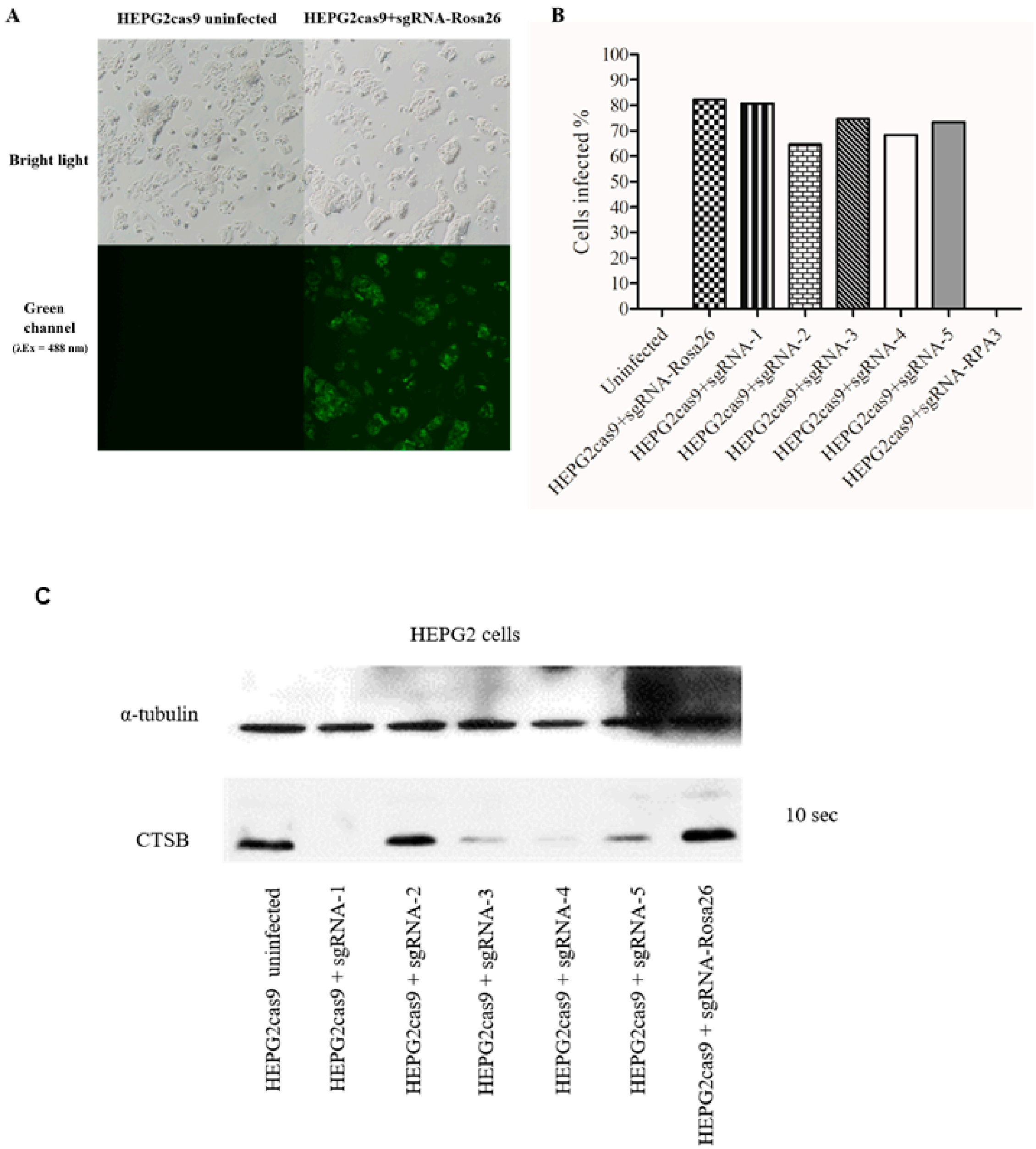

Figure S1. Infection of HEP-G2cas9 cells using lentivirus particles (sgRNA-1 to 5, *Rosa26* and *RPA3*). The cells were infected using the seven different lentiviruses. A- Representative image of HEP-G2cas9 infection using lentivirus particles sgRNA-Rosa26. The same procedure was carried out for all lentivirus produced (sgRNA-1 to 5 for *CTSB* gene and sgRNA-RPA3). The infection was confirmed by GFP expression in inverted fluorescence microscope. The figure

shows the uninfected cells and infected cells with sgRNA-Rosa26 lentivirus expressing GFP under bright light and excitation at 488 nm. B- Infection rate of HEP-G2cas9 cells monitored over the days by GFP fluorescence. Cells infected with different sgRNAs were monitored using flow cytometer MACSQuant® (Miltenyi Biotec). C- Western blotting assay image of HEP-G2cas9 + sgRNAs. HEP-G2cas9 and HEP-G2cas9 + sgRNAs cells were lysed and 50 µg of total protein from each sample were used and the film was exposed for 10 seconds. α-Tubulin protein (60 kDa) was used as total protein load control.
